# Real-time probe-based confocal laser endomicroscopy visualization of dual differentiation features in mixed-type gastric adenocarcinoma

**DOI:** 10.1055/a-2712-8674

**Published:** 2025-10-16

**Authors:** Zhixia Dong, Bo Tian, Shan Wu, Yueqin Qian, Qian Zhuang, Xinjian Wan

**Affiliations:** 1 378725 Digestive Endoscopic Center, Shanghai 6th Peoples Hospital Affiliated to Shanghai Jiao Tong University, Shanghai, China; 2 378725 Shanghai 6th Peoples Hospital Affiliated to Shanghai Jiao Tong University, Shanghai, China; 3 Department of Gastroenterology, Shanghai General Hospital, Shanghai Jiao Tong University School of Medicine, Shanghai, China; 4 Department of Gastroenterology, Shanghai General Hospital of Nanjing Medical University, Shanghai, China

Probe-based confocal laser endomicroscopy (pCLE) has emerged as a revolutionary endoscopic tool, offering real-time, in vivo histological imaging that transforms our understating of complex gastric malignancies 1 2 . We present a case of mixed-type gastric adenocarcinoma in which pCLE played a pivotal role in precise visualization and diagnosis during endoscopic assessment ( Video 1 ).

Probe-based confocal laser endomicroscopy (pCLE) scan of the lesion.Video 1

**Fig. 1:**
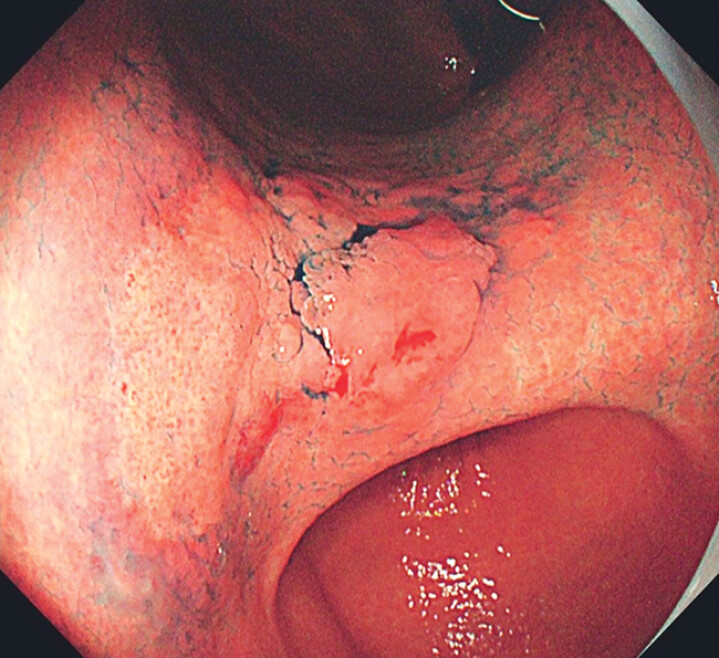
WLE images showed a 3.5×4 cm superficial flat and elevated lesion in the gastric antrum.

**Fig. 2:**
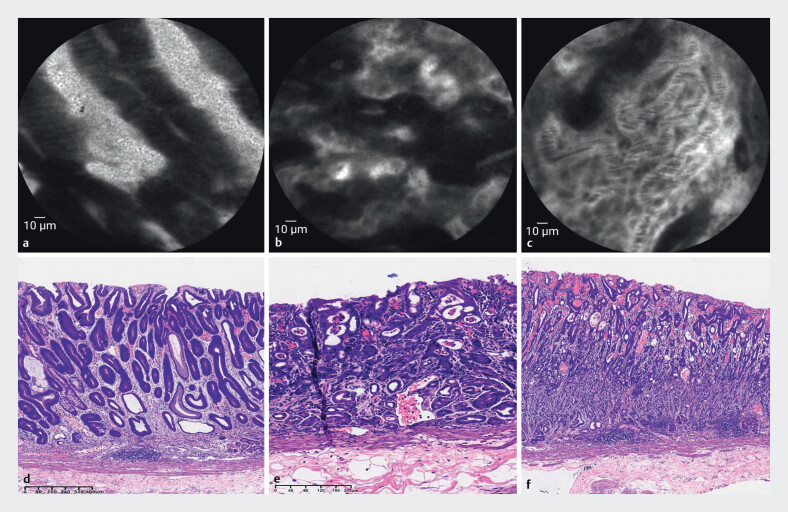
Microstructural imaging characteristics of distinct differentiation components under pCLE. **a** Chaotic glandular arrangement with increased branching and stenosed lumens. **b** Disordered cell arrangement in which normal glandular structure was almost unrecognizable. **c** Development of a dense network of neovascularization. **d** Histopathology showed well-differentiated adenocarcinoma, **e** moderately differentiated adenocarcinoma, and **f** poorly differentiated adenocarcinoma.

A 40-year-old woman with *Helicobacter pylori* infection underwent esophagogastroduodenoscopy. White-light endoscopy (WLE) with indigo carmine chromoendoscopy revealed a 3.5×4 cm erythematous lesion at the gastric angle, featuring a superficial flat-elevated morphology with well-demarcated margins ( Fig. 1 ). It was considered a cancerous lesion according to diagnosis criteria under WLE: 1) well-demarcated border; and 2) irregularity in color/ surface pattern 3 . To further evaluate the lesion, pCLE (1000×magnification, BIOPSEE, Viestar Medical Technology, Wuhan, China) was employed, which revealed distinct microarchitectural patterns: elevated areas showed distorted glands with thickened epithelia ( Fig. 2 **a** ), whereas flat regions exhibited chaotic branching glands and stenosed lumens ( Fig. 2 **b** ). Dedifferentiation was characterized by complete glandular disorganization with dense neovascular networks ( Fig. 2 **c** ). Based on the Miami classification 4 and discriminable glandular structures 5 , mixed adenocarcinoma with dual differentiation was confirmed. Following informed consent, endoscopic submucosal dissection was performed. Real-time pCLE findings correlated perfectly with histopathology: microscopy confirmed both differentiated and undifferentiated components ( Fig. 2 **d** , Fig. 2 **e** ).

This case highlights pCLE's capacity to eliminate diagnostic ambiguity through immediate microscopic pattern recognition, reducing reliance on multiple biopsies. The technology's real-time delineation of heterogeneous tumor components represents a significant advancement toward optical biopsy-driven precision diagnostics for complex malignancies. This case report underscores pCLE's potential to transform endoscopic practice by enabling instant, high-resolution characterization of neoplastic lesions, thereby optimizing therapeutic decision-making.
